# Effect of Ischemic Preconditioning and Postconditioning on Exosome-Rich Fraction microRNA Levels, in Relation with Electrophysiological Parameters and Ventricular Arrhythmia in Experimental Closed-Chest Reperfused Myocardial Infarction

**DOI:** 10.3390/ijms20092140

**Published:** 2019-04-30

**Authors:** Andreas Spannbauer, Denise Traxler, Dominika Lukovic, Katrin Zlabinger, Johannes Winkler, Alfred Gugerell, Péter Ferdinandy, Derek J. Hausenloy, Noemi Pavo, Maximilian Y. Emmert, Simon P. Hoerstrup, Andras Jakab, Mariann Gyöngyösi, Martin Riesenhuber

**Affiliations:** 1 Department of Internal Medicine II, Division of Cardiology, Medical University of Vienna, 1090 Vienna, Austria; andreas.spannbauer@meduniwien.ac.at (A.S.); denise.traxler-weidenauer@meduniwien.ac.at (D.T.); dominika.lukovic@meduniwien.ac.at (D.L.); Katrin.zlabinger@meduniwien.ac.at (K.Z.); Johannes.winkler@meduniwien.ac.at (J.W.); alfred.gugerell@meduniwien.ac.at (A.G.); noemi.pavo@meduniwien.ac.at (N.P.); martin.riesenhuber@meduniwien.ac.at (M.R.); 2 Department of Pharmacology and Pharmacotherapy, Semmelweis University, 1089 Budapest, Hungary; peter.ferdinandy@pharmahungary.com; 3 Pharmahungary Group, 6722 Szeged, Hungary; 4 National Heart Centre Singapore, Singapore 169609, Singapore; d.hausenloy@ucl.ac.uk; 5 Cardiovascular and Metabolic Disorders Program, Duke-National University of Singapore, Singapore 169857, Singapore; 6 The Hatter Cardiovascular Institute, University College London, London WC1E 6HX, UK; 7 Barts Heart Centre, St Bartholomew’s Hospital, London EC1A 7BE, UK; 8 The National Institute of Health Research University College London Hospitals Biomedical Research Centre, London W1T 7DN, UK; 9 Yong Loo Lin School of Medicine, National University Singapore, Singapore 117597, Singapore; 10 Institute for Regenerative Medicine (IREM), University of Zurich, 8952 Zurich, Switzerland; Maximilian.Emmert@usz.ch (M.Y.E.); simon_philipp.hoerstrup@usz.ch (S.P.H.); 11 Department of Cardiovascular Surgery, Charité Universitätsmedizin Berlin, 10117 Berlin, Germany; 12 Department of Cardiothoracic and Vascular Surgery, German Heart Center Berlin, 13353 Berlin, Germany; 13 Center for MR-Research, University Children’s Hospital Zurich, 8032 Zürich, Switzerland; Andras.Jakab@kispi.uzh.ch

**Keywords:** miRNA, extracellular vesicles, exosomes, exosome-rich fraction, ischemic preconditioning, ischemic postconditioning, acute myocardial infarction, cardiac electrophysiology, electrocardiography, ventricular arrhythmia, reperfusion arrhythmia, ischemia-reperfusion injury

## Abstract

We investigated the antiarrhythmic effects of ischemic preconditioning (IPC) and postconditioning (PostC) by intracardiac electrocardiogram (ECG) and measured circulating microRNAs (miRs) that are related to cardiac conduction. Domestic pigs underwent 90-min. percutaneous occlusion of the mid left anterior coronary artery, followed by reperfusion. The animals were divided into three groups: acute myocardial infarction (AMI, *n* = 7), ischemic preconditioning-acute myocardial infarction (IPC-AMI) (*n* = 9), or AMI-PostC (*n* = 5). IPC was induced by three 5-min. episodes of repetitive ischemia/reperfusion cycles (rI/R) before AMI. PostC was induced by six 30-s rI/R immediately after induction of reperfusion 90 min after occlusion. Before the angiographic procedure, a NOGA endocardial mapping catheter was placed again the distal anterior ventricular endocardium to record the intracardiac electrogram (R-amplitude, ST-Elevation, ST-area under the curve (AUC), QRS width, and corrected QT time (QTc)) during the entire procedure. An arrhythmia score was calculated. Cardiac MRI was performed after one-month. IPC led to significantly lower ST-elevation, heart rate, and arrhythmia score during ischemia. PostC induced a rapid recovery of R-amplitude, decrease in QTc, and lower arrhythmia score during reperfusion. Slightly higher levels of miR-26 and miR-133 were observed in AMI compared to groups IPC-AMI and AMI-PostC. Significantly lower levels of miR-1, miR-208, and miR-328 were measured in the AMI-PostC group as compared to animals in group AMI and IPC-AMI. The arrhythmia score was not significantly associated with miRNA plasma levels. Cardiac MRI showed significantly smaller infarct size in the IPC-AMI group when compared to the AMI and AMI-PostC groups. Thus, IPC led to better left ventricular ejection fraction at one-month and it exerted antiarrhythmic effects during ischemia, whereas PostC exhibited antiarrhythmic properties after reperfusion, with significant downregulaton of ischemia-related miRNAs.

## 1. Introduction

Prolonged ischemia is known to cause myocardial necrosis and the loss of myocardial function. Swift reperfusion of the ischemia-affected area of myocardium is currently the gold standard treatment for acute myocardial infarction (AMI). Yet, reperfusion itself can cause additional damage, termed reperfusion injury, which offsets some of the benefits of restoring blood flow. Multiple factors, including oxidative stress, calcium influx, rapid changes to pH levels, or opening of the mitochondrial permeability transition pore (mPTP) likely cause these injuries [1,2,3].

In addition to cellular damage, ischemia and reperfusion can also induce a variety of arrhythmias, including ventricular tachycardia and ventricular fibrillation [4]. During ischemia, the considerable heterogeneity of membrane potentials or the speed of depolarization and refractory periods have been demonstrated within the ischemic myocardium, as well as between the ischemic and non-ischemic regions. Reperfusion seems to amplify these differences by quickly restoring some of these parameters, such as the amplitude of depolarization, without simultaneously restoring others, such as the synchronicity and refractory period, leading to arrhythmias [4,5].

The concept of ischemic preconditioning (IPC) has emerged as one of the most potent protective mechanisms against lethal ischemia/reperfusion (I/R) injury in the heart. Brief episodes of repetitive I/R (rI/R) immediately prior to a longer episode of ischemia significantly reduce infarct size, preserve cardiac function, and decrease the incidence of arrhythmias [6,7,8,9]. However, translating preconditioning to human clinical therapeutic practice has been very challenging due to the limited timespan of IPC’s protective effects and the unpredictable occurrence of human AMI. 

In postconditioning (PostC), reperfusion is immediately followed by multiple short episodes of re-occlusion and the reperfusion of the affected vessel. In theory, this should give the ischemic myocardium more time to re-adapt to normal blood flow, lessening the deleterious effects of the immediate re-oxygenation of the cells and potentially preventing cell membrane rupture. The effect of PostC effect on infarct size initially seemed promising in animal studies, but it has had mixed results in human patients, with a tendency towards smaller or non-persistent reductions in the infarct area when more precise methods of measurement are used [3,10,11,12,13,14,15,16,17,18]. However, PostC has been demonstrated to have a potentially beneficial effect on reperfusion arrhythmias in animal and human studies [9,19,20,21,22,23].

The biomolecular pathways by which PostC and IPC may influence ischemia-induced or reperfusion arrhythmias remain to be elucidated. In this context, microRNAs (miRs) are possible effector molecules. miRs directly interfere with mRNA translation and a single miR copy can repeatedly bind to a large variety of interaction partners, which makes them very potent post-transcriptional gene regulators. While their concentration is the highest within cells, they are also found in the peripheral circulation, where they are most abundant within extracellular vesicles, like exosomes, which protect them from degradation by exonucleases. They may also be involved in remote ischemic conditioning [24]. Their involvement in arrhythmias is an ongoing research topic and only a fraction of their predicted targets that are relevant to cardiac conduction have been experimentally verified.

miR-1 and miR-133 are commonly co-expressed together in myocardial tissue, and their peripheral blood levels are elevated in ischemic heart disease and after myocardial infarction [25]. miR-208a is specifically expressed in cardiomyocytes and its plasma levels correlate with the extent of myocardial injury [26]. miR-21, miR-26, and miR-328 are involved in cardiac remodeling, including fibrosis, neoangiogenesis, and electrical remodeling [27,28,29]. 

Several electrophysiological effects of IPC and PostC have been documented in surface ECGs, including increased ST-segment resolution; reduced QT dispersion, T-amplitude, and corrected QT time (QTc); shortened action potential duration (APD); and, changes in myocardial tissue resistivity [30,31,32,33,34,35]. The NOGA® (Cordis, a Johnson & Johnson company, Irwindale, CA, USA) electromechanical mapping system is capable of simultaneously recording electrical and mechanical data inside the left ventricle (LV), which creates a three-dimensional virtual electroanatomical map and providing information on the viability of the myocardium. In addition, the catheter captures intracardiac ECG, which is much more sensitive than the surface ECG.

We previously showed that rI/R cycles without infarction induce periodic changes in myocardial voltage values, as measured by the endocardial NOGA catheter, which is representative of myocardial viability [36]. In the present study, we investigated intracardiac electrophysiological parameters and the antiarrhythmic effects of IPC and PostC in relation with exosome-bound circulating miRNA levels in a closed chest reperfused AMI animal model. For this purpose, a NOGA mapping catheter was used to record the intracardiac electrical signals at a single stable ischemic left ventricular apical point throughout the entire procedure of myocardial infarction, with or without IPC or PostC.

## 2. Results

**Figure 1:**
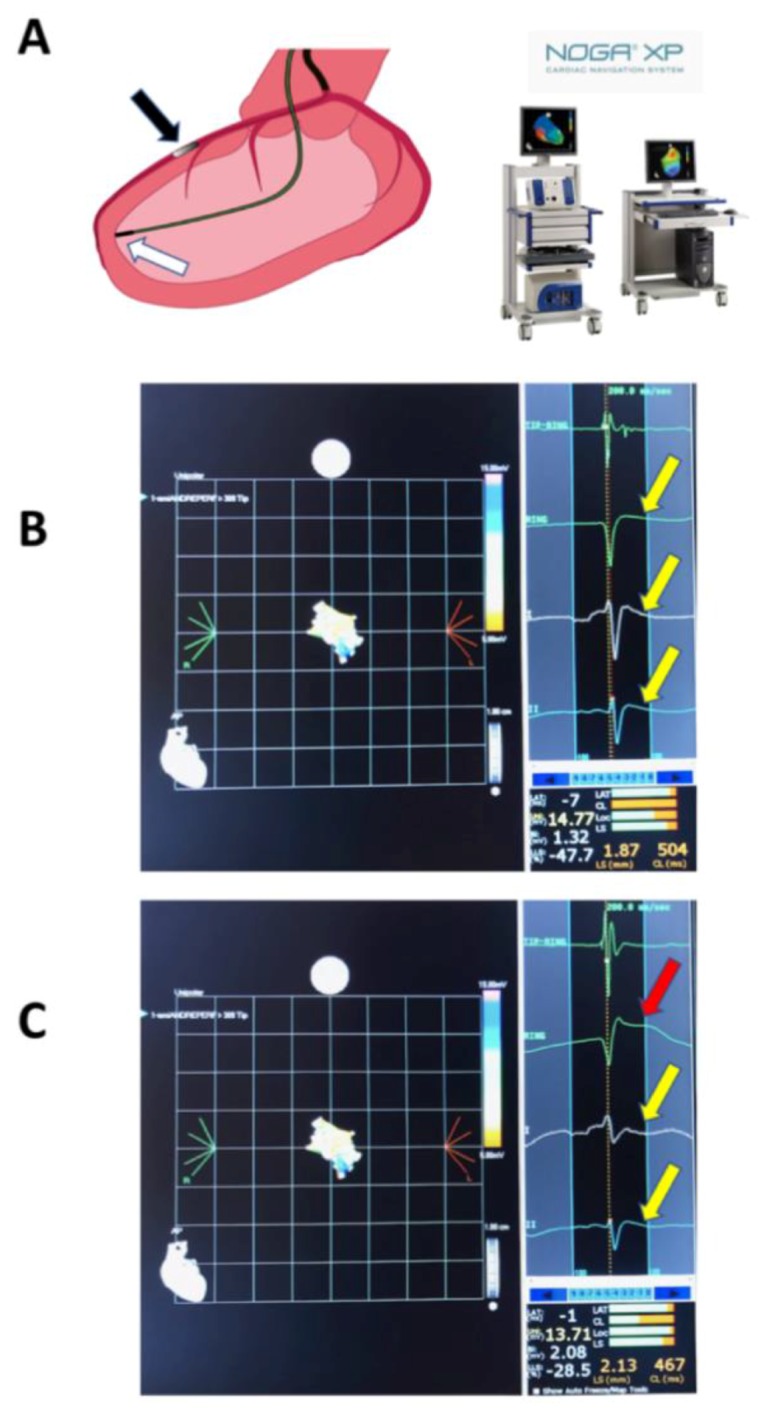
Schematic of the interventional procedure and intracardiac ECG. (**A**) NOGA mapping catheter in the left ventricular cavity touching a fixed endocardial surface point of the ischemic distal anterior wall (white arrow), measuring the endocardial voltage signals and intracardiac ECG; intracoronary balloon in the mid-left anterior descending coronary artery (black arrow), after the origin of the second diagonal branch) (left panel). NOGA equipment enabling online recording of the electrophysiological parameters, and intracardiac and surface ECG (right panel); (**B**) NOGA single point mapping with surface and intracardiac ECGs before the intracoronary balloon occlusion. Note the iso-electric ST-segment in both (yellow arrow); and, (**C**) Intracardiac ST-elevation during intracoronary balloon occlusion (red arrow), and the iso-electric ST-segment in surface ECG (yellow arrow).

One animal in each group died during the coronary occlusion period due to therapy-resistant ventricular arrhythmias. The remaining animals survived the procedure and they were available for follow-up at one-month. Intracardiac ECG was more sensitive for detecting ECG abnormalities when compared to surface ECG, especially ST-changes and QTc interval (Figure 1).

### 2.1. Ischemia-Related ECG Parameters

**Figure 2:**
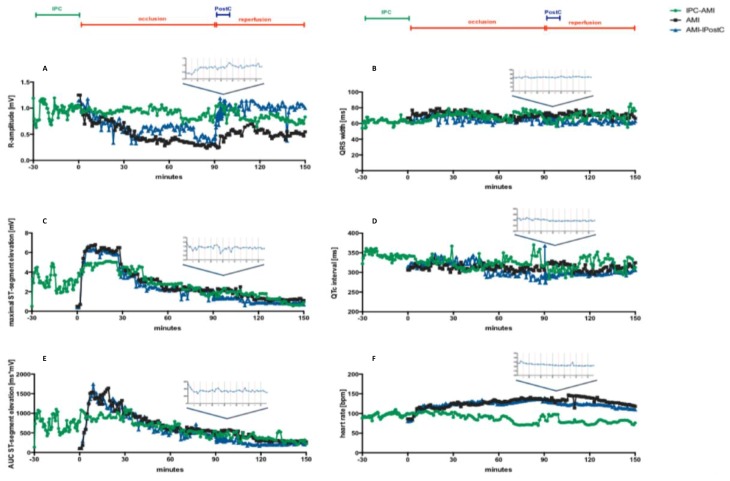
Continuous recording of the ischemia and conduction-related electrophysiological parameters. (**A**) R-amplitude, (**B**) maximum ST-elevation, (**C**) ST area under the curve (AUC-ST), (**D**) QRS width, (**E**) QTc interval, and (**F**) heart rate. Green line: group ischemic preconditioning-acute myocardial infarction (IPC-AMI), black line: group AMI, blue line: group AMI-PostC.

Figure 2 and Table 1 show the development of R-amplitude, ST-elevation and ST-segment area under the curve (AUC), and heart rate. Heart rate increased during the 90-min. coronary occlusion, but to a smaller extent in the IPC-AMI group than in the AMI and AMI-PostC groups. In the IPC-AMI group, the R-amplitude markedly fluctuated during the short rI/R cycles. The difference between ischemic and reperfused R-amplitude seemed to decrease over the three I/R cycles, suggesting ischemic injury of the myocardium, even during short cycles of ischemia (Figure 2). However, in contrast to the AMI and AMI-PostC groups, the IPC-AMI group exhibited fairly stable R-amplitudes during long-term (90 min) ischemia and after reperfusion, suggesting an adaptation of the myocardium to ischemic burden. The significantly higher R-amplitude values in the IPC-AMI group between 10 and 90 min. of coronary occlusion when compared to the other groups support the visual observation (Table 1). After PostC, the R-amplitude of the AMI-PostC animals rapidly increased, indicating the recovery of the ventricular depolarization during the early phase of reperfusion.

IPC led to temporary ST-elevation with recovery during the reperfusion phase of the rI/R cycle. Consistent with adaptive conditioning, the degree of maximum ST-segment elevation during coronary occlusion was the lowest in the IPC-AMI group during the first 30 min. of ischemia (Figure 2, Table 1). PostC led to significant ST-segment elevation resolution when compared to the other groups, indicating microvascular protection. After reperfusion, the ST-segments of all groups returned to baseline values, and no difference between the groups were observed after the 60 min. of reperfusion.

### 2.2. Conduction-Related ECG Parameters

The QRS width remained in the normal range during the whole procedure; therefore, no statistical analysis was performed. The QRS width was also comparable across all three groups (Figure 2). Intracardiac QTc intervals immediately increased during rI/R in the IPC-AMI group, and also in the AMI and AMI-PostC groups after initiating ischemia, and they did not significantly change throughout the experiment, except PostC, which led to a significant temporary decrease in QTc during the PostC rI/R and it remained somewhat lower than in the other groups.

### 2.3. NOGA Electroanatomical Mapping

**Figure 3:**
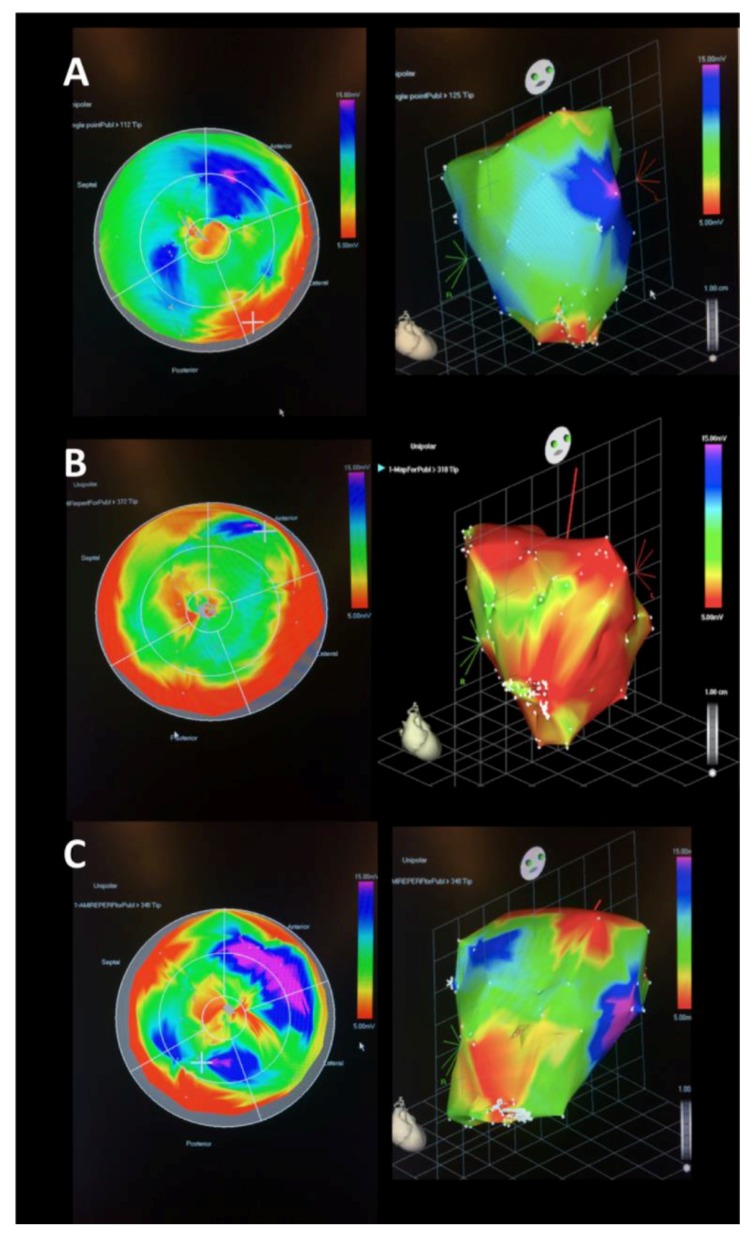
Representative NOGA endocardial surface mapping of unipolar electrical potential (mV) at the end of the AMI procedures. Representative image of an animal in group IPC-AMI (**A**), group AMI, (**B**) and group AMI-PostC (**C**). **Left**: 2D polar maps derived from the 3D maps (Right panel). Voltage values are color-coded, and represent myocardial viability: According to color scale, red: non-viable myocardium, pink and blue viable areas, green and yellow: reduced viability demonstrated. Voltages shown at the marginal edge of the polar map (= close to valvular plane) reflect the naturally low conductivity of the tissue separating atrium and ventricle and are not indicative of ischemic damage. **Right**: 3D representation of the mapped endocardium, with the apex of the ventricle pointing towards the viewer. There is a visual difference in the size of the apical damaged zone in the three groups, with largest viable zone in the IPC-AMI group.

Final NOGA mapping of the LV after the induction of reperfused AMI visually showed a smaller area of infarction in the IPC-AMI group when compared to the AMI and AMI-PostC groups (Figure 3).

### 2.4. Arrhythmia Scores

**Figure 4:**
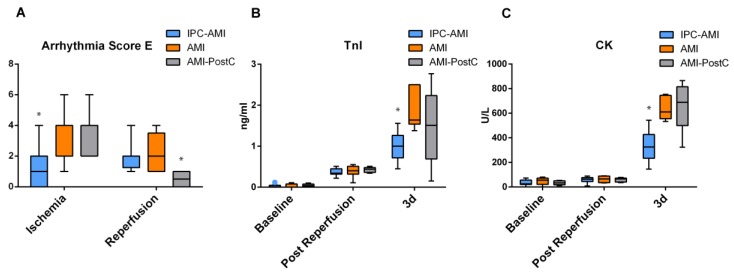
Arrhythmia score and enzymatic infarct size assessment. (**A**) Arrhythmia score, (**B**) Serum level of troponin I (TnI). (**C**) Serum level of creatine kinase (CK). * *p* < 0.05 for IPC-AMI group vs. AMI/AMI-PostC groups during ischemia (A) and at three day (d) follow-up (B and C).

The arrhythmia scores are presented in Figure 4A and Table 2. IPC resulted in a significantly lower incidence of ventricular arrhythmia during ischemia (IPC 1.22 ± 1.30 vs. AMI 2.43 ± 1.99 vs. PostC 2.40 ± 2.07. *p* = 0.041), whereas the AMI-PostC group experienced fewer reperfusion arrhythmias (IPC 1.38 ± 1.30 vs. AMI 1.67 ± 1.03 vs. PostC 0.50 ± 0.58. *p* = 0.033).

### 2.5. Plasma TnI and Creatine Kinase (CK) Values

TnI levels increased immediately at the end of the reperfused AMI period in all groups, and further increased in the three-day follow-up, with significant differences between the IPC-AMI and AMI groups (IPC 0.99 ± 0.34 vs. AMI 1.86 ± 0.43 ng/mL. *p* = 0.021) (Figure 4B). Creatine kinase (CK) levels remained at baseline levels directly after reperfusion, but it increased at three days, with significantly lower levels in the IPC-AMI group than the AMI and AMI-PostC groups (IPC 332.22 ± 114.46 vs. AMI 635.67 ± 87.94 vs. PostC 663.6 ± 182.73 U/L. *p* = 0.001).

### 2.6. qPCR Results

**Figure 5:**
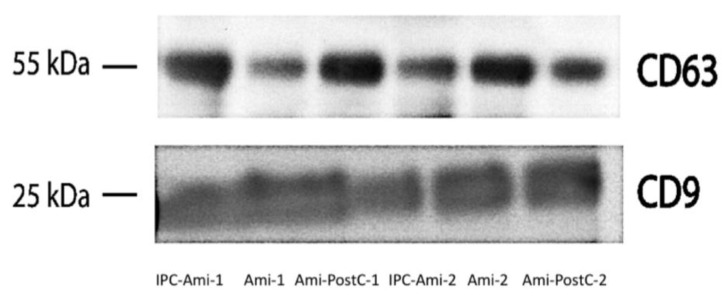
Western Blot of CD9 and CD63. Both proteins are commonly enriched within exosomes and are used to confirm successful exosome isolation.

Western Blot of CD9 and CD63 were used to confirm the enrichment of exosomes in our isolate (Figure 5). 

**Figure 6:**
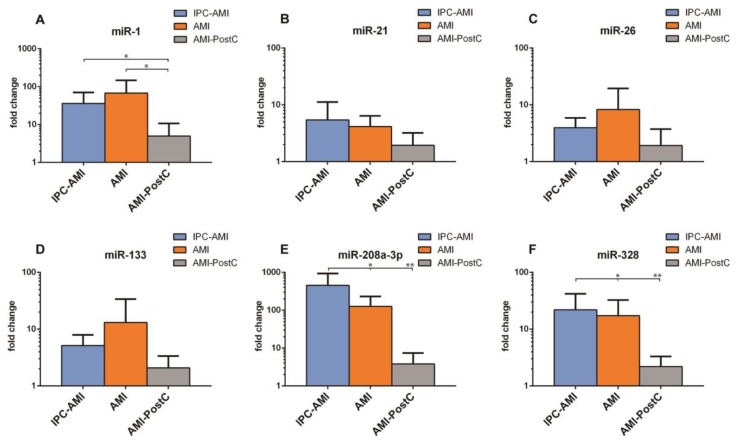
Plasma levels of (**A**) miR-1, (**B**) miR-21, (**C**) miR-26, (**D**) miR-133, (**E**) miR-208a-3p and (**F**) miR-328 in groups. IPC-AMI, AMI and AMI-PostC, quantified by qPCR, immediately after the 60 min reperfusion period. Fold changes (*y* axis) are relative to baseline values of the individual animals. * *p* < 0.05, ** *p* < 0.01.

The further isolation of microRNA from the exosome-rich fraction was successful and all investigated miRs were present in sufficient quantities for analysis (Figure 6). Slightly higher levels of miR-26 (IPC 3.98 ± 1.57 vs. AMI 8.27 ± 9.19 vs. PostC 1.92 ± 1.49 fold increase compared to baseline) and miR-133 (IPC 5.16 ± 2.29 vs. AMI 13.13 ± 16.89 vs. PostC 2.08 ± 1.05) were observed in the AMI group as compared to groups IPC-AMI and AMI-PostC. Significantly lower levels of miR-1 (IPC 36.17 ± 27.98 vs. AMI 67.83 ± 65.57 vs. PostC 4.98 ± 4.72), miR-208 (IPC 454.69 ± 390.15 vs. AMI 126.01 ± 86.32 vs. PostC 3.84 ± 2.93) and miR-328 (IPC 21.88 ± 16.52 vs. AMI 17.34 ± 12.39 vs. PostC 2.20 ± 0.91) were measured in the AMI-PostC group compared to animals in groups AMI and IPC-AMI. 

Across all groups, the magnitude of ST-segment elevation showed a significant positive linear correlation with miR-133 level (*R* = 0.732, *y* = 5.86 + 0.82*x*, *p* = 0.01), while no further association could be observed between electrophysiologic parameters and circulating exosome-enriched miRs. The occurrence of cardiac arrhythmias during ischemia and reperfusion or arrhythmia score did not correlate with the measured circulating miRNAs. 

### 2.7. cMRI Results

Table 3 shows the cMRI data for the three treatment groups. The IPC-AMI group had a significantly reduced infarct size when compared to the AMI-PostC and AMI groups. No significant difference was found between the AMI and AMI-PostC groups in terms of infarct area.

## 3. Discussion

The changes in surface ECG have been used for multiple decades in the diagnosis and grading of myocardial ischemia and infarction. The most important changes relate to ST-elevation, T-waves, and QRS elongation [37,38,39]. In the present study, we investigated the effects of IPC and PostC on intracardiac electrophysiological parameters and ventricular arrhythmias in a porcine model of reperfused myocardial infarction, and related to the circulating exosome-bound miRNAs. The intracardiac ECG measurements allowed for us to more precisely observe these effects than if we had used a surface ECG, because the intracardiac ECG was directly recorded in the ischemic area, whereas a surface ECG is a summation of electrical signals from the whole heart. The novelties of our study are (1) to demonstrate intracardiac electrical parameters of the ischemic area during IPC, and PostC; (2) to present arrhythmia scores during conditioning protocols, (3) to measure circulating levels of miRNAs in the exosome-rich fraction as a final consequence of IPC and PostC in a translational animal model of cardioprotection.

In animal studies, the IPC protocols have consistently been shown to reduce infarct area and increase myocardial salvage after prolonged ischemia [14,40]. However, both animal and human studies have demonstrated mixed effects of IPC on ischemia or reperfusion-induced arrhythmias [41]. In addition, IPC has been shown to reduce ST-elevation, decrease QRS width, and result in the lower grading of ischemia [39,42]. The results of our study are partially consistent with data that are available in the literature, which show a smaller infarct area, lower ST-elevation, lower ST-AUC, higher R-amplitude, and lower ischemia-induced arrhythmia score in IPC when compared to the AMI and AMI-PostC groups. Myocardial ischemia induces the dysfunction of the ATP-sensitive K^+^ (sarcKATP) channel, with prolongation of the effective refractory period (ERP) in the ischemic zone, which sensitizes the myocardium for the induction of ventricular arrhythmias [43]. The attenuation of these effects may be responsible for the IPC-induced antiarrhythmic effect during ischemia observed in our study.

In contrast to the other groups, IPC initially increased the heart rate, but it remained constant thereafter. The lack of increased heart rate during the 90 min. coronary occlusion possibly contributes to the IPC-induced cardioprotective effect by reducing myocardial oxygen consumption. Earlier studies from our group are also consistent with these results.

PostC is a clinically relevant and attractive concept, because it could easily be incorporated into current treatment algorithms for primary percutaneous coronary interventions. Unfortunately, the protective effects have consistently been shown to be smaller than those that are achieved with IPC, and there are substantial disparities between the results of animal and human trials [9,10,11,13,14,16]. In our experiment, PostC did not influence infarct size or ejection fraction, as confirmed by the enzymatic infarct size measurements. Additionally, this finding is in line with our previous experiment which included a higher number of animals and measured the infarct size with cardiac MRI and triphenyl tetrazolium chloride (TTC) staining [18]. There is some evidence from animal and human trials that PostC may be useful in preventing severe reperfusion arrhythmias [8,9,19,20,23]. After PostC, we observed a rapid recovery of the R-amplitude to a similar level as that in the IPC-AMI group, which is a sign of a tendency towards normalization (or pseudo-normalization) of the disturbed myocardial depolarization. Moreover, PostC led to rapid ST-segment resolution in the reperfusion phase. PostC has been shown to exert a beneficial effect on microvascular obstruction and myocardial edema, which may explain its effect on R-amplitude recovery and ST-segment resolution in our study [10,13,18]. Most importantly, the ischemia-induced prolonged QTc was temporarily significantly shortened after PostC, which suggests an antiarrhythmic effect. The prolongation of the QT-interval correlates with the severity of ischemia and increases the chance for lethal arrhythmias, such as torsade de pointes. Several mechanisms have been proposed, including a dysregulation of the slow and rapid repolarizing cardiac potassium currents I_Ks_ and I_Kr_ [44]. Aberrant Na^+^ and Ca^2+^ flow and changes in the cardiac response to catecholamines are included as other contributing mechanisms. 

We chose Arrhythmia Score E because it was most compatible with our study design to quantitatively assess the arrhythmia burden. Scores A, B, and G all include the duration of arrhythmia as a parameter. As we aggressively treated arrhythmias upon onset, aiming for the survival of the animals, these scores would not accurately reflect arrhythmia severity. Score C does not include PVCs, and scores D and F offer less sensitive evaluation than score E (with 0–5 and 0–6 points, respectively, vs. 0–7 points) [45,46].

We chose miR-1, miR-21, miR-26, miR-133, miR-208a and miR-328 for our analysis, because of their versatile and supposed role in cardiac conduction pathophysiology [47] (Table S1). miR-1 and miR-133 affect the ryanodine-receptor-2 (RyR2) through their action on B56α and PP2A, which results in RyR2 hyperphosphorylation, increasing the spontaneous Ca^2+^ spark frequency and ventricular arrhythmia risk [48]. miR-1 also interacts with GJA1 (Connexin 43), while miR-208a is known to target GJA5 (Connexin 40), which are both components of gap junctions. miR-21 and miR-328 are involved in electrical remodeling, with most studies to date pointing towards a connection with atrial fibrillation [27,28]. The finding of a significantly lower fold change of plasma levels of miR-1, miR-208a, and miR-328 in the AMI-PostC group as compared to the IPC-AMI group was unexpected, since these miRNAs are suggested to be in association with infarct burden [26]. Considering the short observation period (max. 180 min), the peak plasma levels of our chosen circulating miRs have likely not yet been reached, which may explain our findings, and also the lack of correlation between the arrhythmia index and levels of miRNAs [49]. In combination with our electrophysiological findings, it is interesting to speculate whether the relative dampening of pro-arrhythmogenic miRs that was observed in our AMI-PostC treatment group might point towards a possible antiarrhythmic benefit of PostC in spite of the failure in reducing infarct size or restoring cardiac function.

### Limitations

Our study has some limitations. First, our group sizes were determined with consideration of the 3 R’s of animal testing (Replace, Reduce, Refine) and that the most significant differences between the groups could be demonstrated. As previously mentioned, the short observation period of our study might not show the full dynamic picture of how the selected circulating miRs are regulated after AMI, but the aim here was to assess them as potential biomarkers for arrhythmias. 

To increase miRNA yield and interpretability of our qPCR we chose to optimize our isolation procedure for miRNA, not for exosome purity. Since most circulating miRNA is thought to be transported within exosomes, further purification would only marginally change miRNA expression patterns, while dramatically decreasing the measurable amount. To reflect this decision, in accordance with recently published position papers on the proper reporting of extracellular vesicle experiments, we have therefore labeled our isolate as “exosome-rich fraction”, instead of as exosomes [50].

Although expression changes in miRNAs in the exosome-rich fraction were not correlated with the arrhythmia score in our study, it cannot be excluded that other miRNAs may contribute to the mechanism of arrhythmia, which might be evaluated with further miRNA-mRNA target network analysis and validations [51,52].

## 4. Materials and Methods

**Figure 7:**
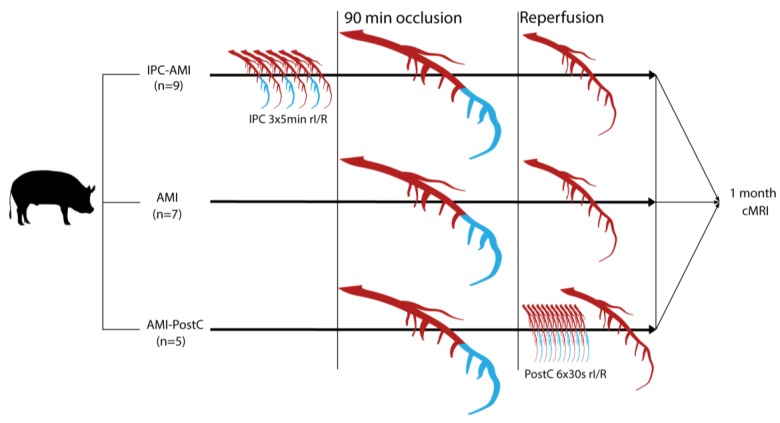
Study design. Domestic pigs were randomized to treatment groups. The mid left anterior descending coronary artery was occluded for 90 min via inflation of a percutaneous coronary balloon, interrupting the perfusion of the distal part of the coronary artery (blue color), while perfused parts of the artery are displayed in red color. After 90min occlusion, the balloon was deflated leading to reperfusion (red color). Ischemic preconditioning (IPC) was performed by three repetitive 5-min cycles of ischemia (blue color of distal coronary artery) /reperfusion (red color of coronary artery) (rI/R), whereas postconditioning (PostC) was elicited by six 30-s cycles of I/R. The animals were then allowed to recover and cardiac magnetic resonance imaging (cMRI) with late enhancement was performed 1 month after the procedure.

The study design is displayed in Figure 7. 

### 4.1. Study Design

The animal experiments were performed at the Institute of Diagnostic Imaging and Radiation Oncology, University of Kaposvar, Hungary (Approval number: SOI/31/26-11/2014). The study was conducted according to the “Position of the American Heart Association on Research Animal Use”. Figure 7 shows the study design. Twenty-one domestic pigs (Sus scrofa, female large whites, 30 ± 2 kg, 3=three months of age) were classified into three groups: reperfused AMI with IPC (IPC-AMI group, *n* = 9), reperfused AMI with PostC (AMI-PostC group, *n* = 5), or reperfused AMI without any cardioprotective procedure (AMI group, *n* = 7). All of the percutaneous procedures were performed under general anesthesia using a femoral access site. Intracardiac ECG was continuously recorded by the NOGA Star electroanatomical mapping catheter and an arrhythmia score was calculated. At the four-week follow-up, LV dimension, function, and infarct size were measured by cardiac magnetic resonance imaging with late enhancement. 

### 4.2. Animal Preparation

One day before AMI induction, the animals received a loading dose of clopidogrel (300 mg) and aspirin (250 mg) per os. After overnight fasting, the pigs were pre-medicated with anesthesia, followed by intratracheal intubation and general anesthesia, as described previously [53]. After placement of the 6F introducer into the right femoral artery, 10.000 IU of unfractionated heparin was given after the placement of the 6F introducer into the right femoral artery. The left femoral artery was also prepared and a 7F introducer placed as access for the intracardiac ECG mapping catheter. The NOGA Star catheter was introduced into the LV and then placed in a stable position in the distal anterior wall. After the stabilization of the catheter position, a 6F coronary guiding catheter was introduced into the left coronary ostium (via right femoral access) and the left coronary arteries visualized by coronary angiogram. A percutaneous balloon catheter (2.5 mm diameter and 10 mm length) was placed after the origin of the second diagonal branch and was inflated with 4–6 atm for 90 min. The occlusion of the distal LAD was verified by angiogram. 

In the preconditioning groups, rI/R was induced by inflation and deflation of an occluding percutaneous balloon catheter in the mid-LAD before the 90 min. occlusion. The IPC-AMI group received three cycles of 5-min. rI/R (5 min. occlusion followed by 5 min. of reperfusion each cycle) [36] immediately prior to the 90-min. occlusion of the mid-LAD, followed by reperfusion. The AMI-PostC group received 90 min. of mid-LAD occlusion, followed by reperfusion. Two minutes within induction of reperfusion, six cycles of 30-sec rI/R were performed by inflation and deflation of the intracoronary balloon, followed by reperfusion. The AMI control group only underwent 90 min. of occlusion of the mid-LAD, followed by reperfusion. 

After the procedure, all of the catheters were removed, the femoral access sutured, anesthesia discontinued, and the pigs returned to their enclosure. After four weeks, cardiac catheterization was repeated to verify the patency of the infarct-related artery.

### 4.3. Intracardiac ECG 

The principles of the NOGA endocardial mapping were reported previously [54]. Briefly, the NOGA Star electroanatomical mapping catheter tip-sensor continuously recorded the intracardiac ECG in real-time. The location of the catheter tip was shown in three-dimensional (3D) according to the ultralow electromagnetic field below the catheter table to ensure a stable location of the NOGA catheter.

An intracardiac electrogram of a single distal anterior point in the ischemic LV was recorded every minute during the procedure, up to 60 min. after final reperfusion, parallel with surface ECG [36,53]. Intracardiac ECG measurements were made every 30 s during IPC and every 10 s during PostC to more accurately capture the changes during these periods. After 60 min. of reperfusion, the entire LV was mapped in randomly selected animals from each group and the voltage of the measured points (viability) was presented in color-coded 2D and 3D maps.

Intracardiac R-amplitude (ventricular depolarization), QRS width (conduction abnormality), QT time (ventricular depolarization and repolarization), maximal ST-elevation, and ST-area under the curve (ST-AUC) (severity and extent of ischemia, respectively) were measured every minute, and the QTc (repolarization disturbance) was calculated according to the Framingham Heart Study adjusted QT time method for parallel measurements of the actual heart rate.

### 4.4. Scoring of Ventricular Arrhythmias

Isolated premature ventricular contraction (PVC), couplets, triplets, ventricular tachycardia (VT), and ventricular fibrillation (VF) were documented during the ischemia, reperfusion, and conditioning periods by continuous intracardiac ECG. Sustained or non-sustained VT was defined as ventricular runs >30 s or <30 s, respectively, with a heart rate >100/min. Idioventricular rhythm was considered to be a slow VT (<100/min), with clear AV dissociation. VF was documented in the case of an irregular heart rhythm with rapid chaotic low voltage electrical impulses in the LV. From the seven original and modified arrhythmia scoring systems [45,46], we chose Arrhythmia Scoring System E, because the other scores contained criteria that were not useful for the present experiments, were not sensitive enough, or that were incompatible with our study design. Briefly, the scoring system was as follows: 0, <20 PVCs; 1, 21–100 PVCs; 2, >100 PVCs or 1–3 episodes of self-limiting VT or both; 3, >3 episodes of self-limiting VT; 4, spontaneous converting VF; 5, 1, or 2 episodes of non-spontaneous converting VF; 6, 3–5 episodes of non-spontaneous converting VF; 7, >5 episodes of non-spontaneous converting VF.

### 4.5. Cardiac cMRI with Late Enhancement

At the one-month follow-up, cardiac MRI with late enhancement (LE) was performed, as described previously [55]. Briefly, ECG-gated cine MRI images were acquired before the injection of 0.05 mmol/kg of gadolinium contrast agent. LE images were acquired 10 min after injection. Cine images were used to determine the end-diastolic volume (EDV), end-systolic volume (ESV), and ejection fraction (EF). The infarct area was determined via measurement of hyperintense regions of myocardium in LE images. The infarct size is given as a percentage of LV mass. All of the MRI analyses were performed by a blinded observer using Segment Software (Medviso AB, Lund, Sweden).

### 4.6. Measurements of Ischemia Biomarkers Troponin I and Creatine Kinase

Serum levels of troponin I (TnI) and creatine kinase (CK) were measured at baseline before the procedure, immediately after the 60 min of reperfusion, and at three days using porcine-specific ELISA kits (Cloud Clone Corp, Houston, TX, USA) and the protocols that were recommended by the manufacturer. 

### 4.7. Isolation of Exosome-Rich Fraction

EDTA plasma samples were taken before the first occlusion and immediately after 60 min. of reperfusion. Plasma was prepared for exosome enrichment by progressive centrifugation steps of 1200× *g* for 10 min., 1800× *g* for 10 min., and 10,000× g for 20 min. The resulting plasma was then filtered through 0.2 μm syringe filters. 500 µL were then suspended in 10 mL PBS and ultra-centrifuged at 130,000× *g* for 180 min. (Rotor: SW 40 Ti, 27,100 rpm) to maximize the RNA yield. Exosome-rich fraction pellets were resuspended in 200 µL PBS. Western Blot of CD63 and CD9 verified the enrichment of exosomes. 

### 4.8. miR Isolation and qPCR

miRs were isolated from 200 µL exosome-rich fraction in PBS, equivalent to 500 µL EDTA-plasma, using QIAGEN miRNeasy Serum/Plasma kits, reverse transcribed using QIAGEN miScript RT kit and qPCR was performed using miScript SYBR® Green PCR Kit, according to manufacturers’ protocols. Conduction-related miR-1, miR-21, miR-26, miR-133a, miR-208a-3p, and miR-328 were quantified. The fold changes were normalized using ce-miR-39 Spike-in-Control of the QIAGEN Serum/Plasma kit. Fold change after reperfusion was calculated while using pre-intervention expression levels as baseline.

### 4.9. Statistical Analysis 

Continuous data were presented as mean and standard deviation, and categorical data as numbers and percentages. The electrophysiological parameters were averaged for select time intervals in each group. Differences between groups were calculated by the analysis of variance (ANOVA) and Tukey *post-hoc* test for select time points during the experiment: baseline, 0–10 min, 10–20 min, 20–30 min, 30–60 min, and 60–90 min during LAD occlusion and 0–10 min, 10–20 min, 20–30 min, and 30–60 min after final reperfusion. The evolution of parameters as means were visualized using *x*,*y*-plots. In all cases, a difference with *p* < 0.05 was considered to be significant. Statistical analyses were performed using SPSS 23.0 (IBM Corp., Armonk, NY, USA) and GraphPad Prism 6.0 (La Jolla, CA, USA) software.

## 5. Conclusions

In conclusion, our study has shown that IPC and PostC exert different antiarrhythmic effects, as manifested by changes in intracardiac ECG signals. IPC protects the myocardium against life-threatening ischemia-induced arrhythmias during coronary occlusion, as shown by diminished ST-elevation, the partial preservation of the depolarizing signal R-wave, and the inhibition of sinus tachycardia, which translated to a decreased infarct size and improved ventricular function after myocardial infarction. PostC did not significantly reduce infarct size, but we observed a recovery of the R-amplitude, mitigation of the ST-segment elevation, a temporary decrease in ischemia and reperfusion injury-induced prolonged QTc, and decrease of arrhythmia during the reperfusion phase. The measurement of circulating exosome-bound miRNAs did not result in conclusive statements regarding association with antiarrhythmic or cardioprotective effects of IPC. However, cardiac ischemia-related miRNAs plasma levels were systematically lower in AMI-PostC animals, suggesting a role in microvascular protection and the prevention of reperfusion-induced arrhythmias. 

## Figures and Tables

**Table 1:** Evolution of ischemia and conduction-related electrophysiological parameters during cardioprotective interventions.

R-Amplitude (mV)					
	Time	IPC-AMI (*n* = 9)	AMI-PostC (*n* = 5)	AMI (*n* = 7)	*p*-Value
Baseline		1.26 ± 0.54	1.22 ± 0.47	1.27 ± 0.22	n.s.
IPC	Occlusion 3 × 5min. (pooled)	1.01 ± 0.58			
	Reperfusion 3 × 5min. (pooled)	1.27 ± 0.83			
Occlusion	1–10 min	1.07 ± 0.50	1.05 ± 0.43	1.02 ± 0.88	n.s.
	10–20 min	1.04 ± 0.54 *	0.86 ± 0.36 *	0.83 ± 0.33	0.007
	20–30 min	1.06 ± 0.50 *	0.83 ± 0.52 *	0.72 ± 0.23 †	<0.001
	30–60min	1.19 ± 0.80 *,†	0.66 ± 0.34 *,°	0.48 ± 0.25 †,°	<0.001
	60–90 min	1.03 ± 0.65 *,†	0.57 ± 0.23 *	0.44 ± 0.37 †	<0.001
PostC	Reperfusion 6 × 30sec (pooled)		0.92 ± 0.42		
	Occlusion 6 × 30sec (pooled)		0.98 ± 0.42		
Reperfusion	1–10 min. rep	1.11 ± 0.45 †	1.10 ± 0.43 °	0.47 ± 0.28 †,°	<0.001
	10–20 min. rep	1.09 ± 0.57 †	1.12 ± 0.35 °	0.68 ± 0.46 †,°	<0.001
	20–30 min. rep	1.01 ± 0.68 †	1.06 ± 0.34 °	0.75 ± 0.53 †,°	0.013
	30–60 min. rep	0.98 ± 0.61 †	1.06 ± 0.37 °	0.61 ± 0.36,°	<0.001
Maximal ST-Segment Elevation (mV)					
	Time	IPC-AMI (*n* = 9)	AMI-PostC (*n* = 5)	AMI (*n* = 7)	*p*-Value
Baseline		0.66 ± 0.38	0.50 ± 0.33	0.50 ± 0.18	n.s.
IPC	Occlusion 3 × 5min. (pooled)	3.47 ± 1.41			
	Reperfusion 3 × 5min. (pooled)	2.71 ± 1.10			
Occlusion	1–10 min	4.19 ± 1.66 *	4.82 ± 2.19 *	5.53 ± 2.09	<0.001
	10–20 min	4.59 ± 1.35 *,†	5.78 ± 1.32 *	6.24 ± 1.33 †	<0.001
	20–30 min	4.19 ± 1.32 *,†	5.13 ± 1.46 *	5.67 ± 1.73 †	<0.001
	30–60min	3.71 ± 1.62 *,†	2.98 ± 1.05 *	3.21 ± 1.33 †	<0.001
	60–90 min	2.42 ± 1.10 *,†	2.06 ± 0.70 *	2.20 ± 0.76 †	0.003
PostC	Reperfusion 6x30sec (pooled)		1.42 ± 0.41		
	Occlusion 6x30sec (pooled)		1.37 ± 0.41		
Reperfusion	1–10 min. rep	2.21 ± 1.09 *	1.15 ± 0.42 *,°	2.03 ± 0.51 °	<0.001
	10–20 min. rep	1.93 ± 0.71 *	0.92 ± 0.54 *,°	1.78 ± 0.67 °	<0.001
	20–30 min. rep	1.59 ± 0.70 *	0.87 ± 0.22 *,°	1.42 ± 0.60 °	<0.001
	30–60 min. rep	1.19 ± 0.79	0.88 ± 0.26	1.12 ± 0.42	n.s.
QTc (ms)					
	Time	IPC-AMI (*n* = 9)	AMI-PostC (*n* = 5)	AMI (*n* = 7)	*p*-Value
Baseline		309 ± 85	309 ± 31	311 ± 18	n.s.
IPC	Occlusion 3 × 5min. (pooled)	319 ± 92			
	Reperfusion 3 × 5min. (pooled)	318 ± 101			
Occlusion	1–10 min	327 ± 91	316 ± 33	312 ± 23	n.s.
	10–20 min	333 ± 97	320 ± 46	322 ± 41	n.s.
	20–30 min	330 ± 104	325 ± 34	331 ± 66	n.s.
	30–60min	320 ± 97	320 ± 38	323 ± 60	n.s.
	60–90 min	324 ± 96	318 ± 47	325 ± 54	n.s.
PostC	Reperfusion 6 × 30sec (pooled)		310 ± 84		
	Occlusion 6 × 30sec (pooled)		309 ± 84		
Reperfusion	1–10 min. rep	320 ± 96 *	305 ± 91 *,°	326 ± 37 °	0.037
	10–20 min. rep	321 ± 97	305 ± 82	325 ± 50	n.s.
	20–30 min. rep	320 ± 113	308 ± 67	325 ± 78	n.s.
	30–60 min. rep	319 ± 102	305 ± 73	326 ± 52	n.s.
Heart rate (bpm)					
	Time	IPC-AMI (*n* = 9)	AMI-PostC (*n* = 5)	AMI (*n* = 7)	p-value
Baseline		87 ± 21	80 ± 34	85 ± 20.08	n.s.
IPC	Occlusion 3 × 5min. (pooled)	96 ± 33			
	Reperfusion 3 × 5min. (pooled)	91 ± 24			
Occlusion	1–10 min	98 ± 32	103 ± 27	104 ± 23	n.s.
	10–20 min	103 ± 44 *,†	117 ± 6 *	115 ± 25 †	0.019
	20–30 min	96 ± 69 *,†	119 ± 7 *	115 ± 19 †	0.015
	30–60min	89 ± 27 *,†	127 ± 7 *	121 ± 21 †	<0.001
	60–90 min	84 ± 23 *,†	127 ± 12 *	124 ± 25 †	<0.001
PostC	Reperfusion 6 × 30sec (pooled)		125 ± 13		
	Occlusion 6 × 30sec (pooled)		124 ± 12		
Reperfusion	1–10 min. rep	92 ± 20 *,†	119 ± 10 *	125 ± 23 †	<0.001
	10–20 min. rep	84 ± 24 *,†	119 ± 10 *	126 ± 25 †	<0.001
	20–30 min. rep	84 ± 18 *,†	120 ± 7 *	131 ± 32 †	<0.001
	30–60 min. rep	78 ± 18	114 ± 7	125 ± 22	<0.001

Data are presented as mean ± SD. *p*-Values were determined by one-way ANOVA supplemented with Tukey *post-hoc* test of the three groups during the specified time period. † *p* < 0.05 between groups IPC-AMI vs AMI; * *p* < 0.05 between groups IPC-AMI vs AMI-PostC; ° *p* < 0.05 between groups AMI-PostC vs AMI; n.s.—nonsignificant.

**Table 2:** Arrhythmia Score E. The numbers in the rows correspond to the number of animals that reached the arrhythmia score of that particular column.

Arrhythmia Score E		Score (0–6)							
		0	1	2	3	4	5	6	IVR
Ischemia	IPC-AMI	3	3	2		1			
	AMI		1	3	1	1		1	
	AMI-PostC		4				1		
Reperfusion	IPC-AMI	3	6						5
	AMI		4	1	1	1			3
	AMI-PostC	3	2						2

Score 0 (0-20 PVCs); Score 1 (21 – 100 PVCs); Score 2 (>100 PVCs and/or 1-2 self-limited VTs); Score 3 (>3 self-limited VTs); Score 4 (spontaneously converting VFs); Score 5 (1-2 non-converting VFs); Score 6 (3-5 non-converting VFs); PVC – Premature Ventricular Contraction, VT – Ventricular Tachycardia, VF – Ventricular Fibrillation, IVR – Idioventricular Rhythm.

**Table 3:** MRI follow-up one-month after reperfused AMI with or without IPC or PostC.

Variable	IPC-AMI (*n* = 8)+	AMI-PostC (*n* = 4)+	AMI (*n* = 6)+	*p*-Value
Infarct size of LV (%)	17.0 ± 7.1 *	24.5 ± 3.8	25.2 ± 5.1	0.047
LVEF (%)	40.9 ± 4.2	37.2 ± 5.5	35.2 ± 4.6	n.s.
LVEDVI (mL)	66.3 ± 6.0	70.3 ± 12.4	64.5 ± 7.8	n.s.
LVESVI (mL)	39.2 ± 4.8	44.1 ± 9.1	41.7 ± 4.4	n.s.

Mean±SD; + one animal of each group died during procedure, thus, numbers represent the surviving animals up to one-month follow-up. LV—left ventricle; LVEF—left ventricular ejection fraction; LVEDVI—left ventricular end-diastolic volume index; LVESVI—left ventricular end-systolic volume index., n.s.—non-significant; * *p* < 0.05 between IPC-AMI vs AMI/AMI-PostC.

## Supplementary Materials

Supplementary materials can be found at https://www.mdpi.com/1422-0067/20/9/2140/s1.
