# Supplementary material for: Effect of Ischemic Preconditioning and Postconditioning on Exosome-Rich Fraction microRNA Levels, in Relation with Electrophysiological Parameters and Ventricular Arrhythmia in Experimental Closed-Chest Reperfused Myocardial Infarction
Source: Int J Mol Sci. 2019 Apr 30;20(9):2140. doi: 10.3390/ijms20092140 (PMC6540096; doi:10.3390/ijms20092140)
Supplement: Supplementary file 1 [file ijms-20-02140-s001.pdf]

Effect of Ischemic Preconditioning and Postconditioning on exosome-rich fraction microRNA Levels, in Relation with Electrophysiological Parameters and Ventricular Arrhythmia in Experimental Closed-Chest Reperfused Myocardial Infarction

Andreas Spannbauer MD, Denise Traxler MD, Dominika Lukovic MSc PhD, Katrin Zlabinger MSc, Johannes Winkler MSc PhD, Alfred Gugerell MSc PhD, Péter Ferdinandy MD PhD MBA, Derek J. Hausenloy MBChB PhD, Noemi Pavo MD PhD, Maximilian Y. Emmert MD PhD, Simon P. Hoerstrup MD PhD, Andras Jakab MD PhD, Mariann Gyöngyösi MD PhD and Martin Riesenhuber MD

Supplementary information

Table S1. Measured miRs and cardiac-conduction related effects. This table shows an overview of our measured miRs, their known dynamic in I/R, the dynamics between our groups and a more detailed list of conduction related effects, gene targets (brackets denote the coded protein) and other known functions of these miRs.

**miR**	**Regulation in I/R**	**Dynamic in our study**	**Conduction related effects**	**Cardiac Conduction related gene target**	**Other functions**
miR-1	↑ in plasma	↑ ↑ IPC<br>↑ ↑ ↑ AMI<br>↑ PostC	↑ Ca2+ release, increased excitability,	B56α (RyR2)[1,2]<br>GJA1 (Cx43)[3,4]<br>KCNJ2 (Kir2.1)[3]	CTGF, TGFβ1, Calmodulin, Mef2a, Anti-fibrotic [5,6]<br>Bcl-2 Promotes apoptosis [7,8]
miR-21	↑ in plasma	↑ ↑ ↑ IPC<br>↑ ↑ AMI<br>↑ PostC	↓ L-Type Calcium Current in Atria[9]		Spry1 Pro-fibrotic[10]<br>PDCD4 Anti-Apoptotic [11]
miR-26	↑ in plasma	↑ ↑ IPC<br>↑ ↑ ↑ AMI<br>↑ PostC	↓ inward rectifier K+ channels (↓ AF)	KCNJ2 (Kir2.1)[12]	GATA4, PLC1b<br>Pro-fibrotic, anti-angiogenic [13,14]
miR-133	↑ in plasma	↑ ↑ IPC<br>↑ ↑ ↑ AMI<br>↑ PostC	↑ Ca2+ release<br>↓ transient outward K+ current, I(to,f)[15]<br>↓ I_{Kr}	B56α (RyR2)[1]<br>PP2A (RyR2)<br>ERG	Calcineurin<br>Anti-fibrotic[16,17]<br>Caspase 9<br>Anti-apoptotic

miR-208a	↑ in plasma	↑ ↑ ↑ IPC<br>↑ ↑ AMI<br>↑ PostC	↓ conduction speed	GJA5	GATA4, Connexin40, THRAP3, Myostatin<br>Pro-hypertrophy, Necessary for atrial conduction [18]
miR-328	↑ in plasma	↑ ↑ ↑ IPC<br>↑ ↑ AMI<br>↑ PostC	Adverse electrical remodeling (↑ AF) [19]<br>↓ L-Type Calcium Channels	CACNA1C	

Supplementary References

1. Belevych, A.E.; Sansom, S.E.; Terentyeva, R.; Ho, H.T.; Nishijima, Y.; Martin, M.M.; Jindal, H.K.; Rochira, J.A.; Kunitomo, Y.; Abdellatif, M., et al. MicroRNA-1 and -133 increase arrhythmogenesis in heart failure by dissociating phosphatase activity from RyR2 complex. *PloS one* **2011**, *6*, e28324, doi:10.1371/journal.pone.0028324.
2. Terentyev, D.; Belevych, A.E.; Terentyeva, R.; Martin, M.M.; Malana, G.E.; Kuhn, D.E.; Abdellatif, M.; Feldman, D.S.; Elton, T.S.; Gyorke, S. miR-1 overexpression enhances Ca(2+) release and promotes cardiac arrhythmogenesis by targeting PP2A regulatory subunit B56alpha and causing CaMKII-dependent hyperphosphorylation of RyR2. *Circ Res* **2009**, *104*, 514-521, doi:10.1161/circresaha.108.181651.
3. Yang, B.; Lin, H.; Xiao, J.; Lu, Y.; Luo, X.; Li, B.; Zhang, Y.; Xu, C.; Bai, Y.; Wang, H., et al. The muscle-specific microRNA miR-1 regulates cardiac arrhythmogenic potential by targeting GJA1 and KCNJ2. *Nature medicine* **2007**, *13*, 486-491, doi:10.1038/nm1569.
4. Curcio, A.; Torella, D.; Iaconetti, C.; Pasceri, E.; Sabatino, J.; Sorrentino, S.; Giampa, S.; Micieli, M.; Polimeni, A.; Henning, B.J., et al. MicroRNA-1 downregulation increases connexin 43 displacement and induces ventricular tachyarrhythmias in rodent hypertrophic hearts. *PloS one* **2013**, *8*, e70158, doi:10.1371/journal.pone.0070158.
5. Karakikes, I.; Chaanine, A.H.; Kang, S.; Mukete, B.N.; Jeong, D.; Zhang, S.; Hajjar, R.J.; Lebeche, D. Therapeutic cardiac-targeted delivery of miR-1 reverses pressure overload-induced cardiac hypertrophy and attenuates pathological remodeling. *Journal of the American Heart Association* **2013**, *2*, e000078, doi:10.1161/jaha.113.000078.
6. Ikeda, S.; He, A.; Kong, S.W.; Lu, J.; Bejar, R.; Bodyak, N.; Lee, K.H.; Ma, Q.; Kang, P.M.; Golub, T.R., et al. MicroRNA-1 negatively regulates expression of the hypertrophy-associated calmodulin and Mef2a genes. *Molecular and cellular biology* **2009**, *29*, 2193-2204, doi:10.1128/mcb.01222-08.
7. Tang, Y.; Zheng, J.; Sun, Y.; Wu, Z.; Liu, Z.; Huang, G. MicroRNA-1 regulates cardiomyocyte apoptosis by targeting Bcl-2. *International heart journal* **2009**, *50*, 377-387.
8. Pan, Z.; Sun, X.; Ren, J.; Li, X.; Gao, X.; Lu, C.; Zhang, Y.; Sun, H.; Wang, Y.; Wang, H., et al. miR-1 exacerbates cardiac ischemia-reperfusion injury in mouse models. *PloS one* **2012**, *7*, e50515, doi:10.1371/journal.pone.0050515.

9. Barana, A.; Matamoros, M.; Dolz-Gaitón, P.; Pérez-Hernández, M.; Amorós, I.; Núñez, M.; Sacristán, S.; Pedraz, Á.; Pinto, Á.; Fernández-Avilés, F., et al. Chronic Atrial Fibrillation Increases MicroRNA-21 in Human Atrial Myocytes Decreasing L-Type Calcium Current. *Circulation: Arrhythmia and Electrophysiology* **2014**, *7*, 861-868, doi:10.1161/CIRCEP.114.001709.
10. Thum, T.; Gross, C.; Fiedler, J.; Fischer, T.; Kissler, S.; Bussen, M.; Galuppo, P.; Just, S.; Rottbauer, W.; Frantz, S., et al. MicroRNA-21 contributes to myocardial disease by stimulating MAP kinase signalling in fibroblasts. *Nature* **2008**, *456*, 980-984, doi:10.1038/nature07511.
11. Cheng, Y.; Zhu, P.; Yang, J.; Liu, X.; Dong, S.; Wang, X.; Chun, B.; Zhuang, J.; Zhang, C. Ischaemic preconditioning-regulated miR-21 protects heart against ischaemia/reperfusion injury via anti-apoptosis through its target PDCD4. *Cardiovascular research* **2010**, *87*, 431-439, doi:10.1093/cvr/cvq082.
12. Luo, X.; Pan, Z.; Shan, H.; Xiao, J.; Sun, X.; Wang, N.; Lin, H.; Xiao, L.; Maguy, A.; Qi, X.Y., et al. MicroRNA-26 governs profibrillatory inward-rectifier potassium current changes in atrial fibrillation. *The Journal of clinical investigation* **2013**, *123*, 1939-1951, doi:10.1172/jci62185.
13. Han, M.; Yang, Z.; Sayed, D.; He, M.; Gao, S.; Lin, L.; Yoon, S.; Abdellatif, M. GATA4 expression is primarily regulated via a miR-26b-dependent post-transcriptional mechanism during cardiac hypertrophy. *Cardiovascular research* **2012**, *93*, 645-654, doi:10.1093/cvr/cvs001.
14. Heineke, J.; Auger-Messier, M.; Xu, J.; Oka, T.; Sargent, M.A.; York, A.; Klevitsky, R.; Vaikunth, S.; Duncan, S.A.; Aronow, B.J., et al. Cardiomyocyte GATA4 functions as a stress-responsive regulator of angiogenesis in the murine heart. *The Journal of clinical investigation* **2007**, *117*, 3198-3210, doi:10.1172/JCI32573.
15. Matkovich, S.J.; Wang, W.; Tu, Y.; Eschenbacher, W.H.; Dorn, L.E.; Condorelli, G.; Diwan, A.; Nerbonne, J.M.; Dorn, G.W., 2nd. MicroRNA-133a protects against myocardial fibrosis and modulates electrical repolarization without affecting hypertrophy in pressure-overloaded adult hearts. *Circ Res* **2010**, *106*, 166-175, doi:10.1161/circresaha.109.202176.
16. Dong, D.L.; Chen, C.; Huo, R.; Wang, N.; Li, Z.; Tu, Y.J.; Hu, J.T.; Chu, X.; Huang, W.; Yang, B.F. Reciprocal repression between microRNA-133 and calcineurin regulates cardiac hypertrophy: a novel mechanism for progressive cardiac hypertrophy. *Hypertension (Dallas, Tex. : 1979)* **2010**, *55*, 946-952, doi:10.1161/hypertensionaha.109.139519.
17. Care, A.; Catalucci, D.; Felicetti, F.; Bonci, D.; Addario, A.; Gallo, P.; Bang, M.L.; Segnalini, P.; Gu, Y.; Dalton, N.D., et al. MicroRNA-133 controls cardiac hypertrophy. *Nature medicine* **2007**, *13*, 613-618, doi:10.1038/nm1582.
18. Callis, T.E.; Pandya, K.; Seok, H.Y.; Tang, R.H.; Tatsuguchi, M.; Huang, Z.P.; Chen, J.F.; Deng, Z.; Gunn, B.; Shumate, J., et al. MicroRNA-208a is a regulator of cardiac hypertrophy and conduction in mice. *The Journal of clinical investigation* **2009**, *119*, 2772-2786, doi:10.1172/jci36154.
19. Lu, Y.; Zhang, Y.; Wang, N.; Pan, Z.; Gao, X.; Zhang, F.; Zhang, Y.; Shan, H.; Luo, X.; Bai, Y., et al. MicroRNA-328 contributes to adverse electrical remodeling in atrial fibrillation. *Circulation* **2010**, *122*, 2378-2387, doi:10.1161/circulationaha.110.958967.
